# Cryopreservation of orchid seeds through rapid and step freezing methods

**DOI:** 10.12688/f1000research.13622.1

**Published:** 2018-02-20

**Authors:** Marco Cerna, Paulina Valdivieso, Rino Cella, Bence Mátyás, Cristina Aucapiña

**Affiliations:** 1 Carrera de Biotecnología de los Recursos Naturales, Universidad Politécnica Salesiana, Quito, Ecuador; 2 Jardín Botanico “Orquídeas de Sarina”, Quito, Ecuador; 3 Laboratorio de biologia molecular vegetal, University of Pavia, Pavia, Italy; 4 Grupo de Investigación Mentoria y Gestión del Cambio, Universidad Politécnica Salesiana, Cuenca, Ecuador

**Keywords:** Orchidaceae, Epidendrum, Sobralia, seeds, cryoconservation, liquid nitrogen, Tetrazolium.

## Abstract

Ecuador has a great variety of climatic regions that potentiate biodiversity. The family *Orchidaceae *constitutes one of the most important of the country, having identified about 4032 species with a high degree of endemism, therefore the development and research of alternative methods of storage and conservation of species is a strategy of primary interest for researchers and for society in general. In cryopreservation, temperatures reach below -190°C in order to paralyze the chemical reactions and keep the plant material viable for long periods. The present research focuses on the development of protocols for cryopreservation of seeds, aimed at the preservation of biodiversity, focusing on the family *Orchidaceae*, for the subsequent generation of a seed bank. The assays were performed on seeds of *Epidendrum quitensium, Sobralia rosea*, and *Epidendrum anderssonii*. Two freezing rates were tested: rapid freezing at -196°C; and step freezing at -22°C, -60°C to 196°C, further analyzed four combinations from Dimethylsulfoxide DMSO, glycerol and sucrose (DMSO 1M; DMSO 1M + glycerol 1M; DMSO 1M + sucrose 1M; DMSO 1M + glycerol 0,5M + sucrose 0,5M). The best results were obtained both in rapid and stepped freezing without the use of cryo-protective substances, by introducing the seeds directly into liquid nitrogen. Species of the genus *Epidendrum *presented a more efficient response in comparison to *Sobralia*. The viability of the seeds was evaluated by the tetrazolium test.

## Introduction

The Republic of Ecuador is located on the South American continent. From north to south the country is crossed by the Andes mountain range and has four climatic regions: Coast, Andes, Amazon and the Insular region ^1^. Its position in the middle of the world, the luminous intensity, the ocean currents and the different altitudes produce 82 types of ecosystems (see Ministry of Environment document on ecosystems in Ecuador) There is a great variety of climatic regions that have an important effect in the diversification of plant formations ^2^. Concerning the *Orchidaceae* family, in Ecuador as of 2010, 4032 species of orchids have been identified, of which 1714 (42.5%) are endemic ^3^; 4.5% of the orchids of the planet are found in Ecuador. Seed banks allow the conservation of the biodiversity *ex situ* and prioritize species used for food, medicine and those in danger of extinction. *Orchidaceae* is a large family with many endangered species and all of them are included in the Convention on International Trade in Endangered Species of Wild Fauna and Flora (CITES) I and II ^4^. Cryopreservation is an efficient strategy to safeguard these species, but unfortunately, orchid seeds have short lifetimes ^5^; the longevity depends on the moisture content and storage temperature, so it is necessary to experiment with efficient storage systems for each species ^5^. The advantages of cryopreservation are: storage for an indefinite period, genetic stability of the individuals, reduced infrastructure, can have independent energy and the stored genetic material does not require manipulation ^6^.

Therefore, the objective of this research was to define protocols for cryopreservation of orchid seeds, in order to install a seed bank that promotes the conservation of vulnerable species.

## Methods

### Collection of biological material

The collection of plant material was made through the authorization of the Ministry of Environment of Ecuador No. 17-2011- Investigación-B- DPMS/MAE,FloraX, N0. 08 *−*2013 *−*0869 *−I C _F_AU−F LO−DAPI −UNO−MAE* and the Botanical Garden "Orquídeas de Sarina" patent No. 006-2015- FLO-DPAP- MA.

**Figure 1.**
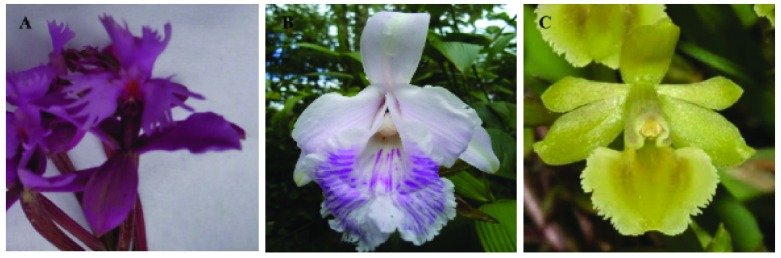
Orchids used for cryopreservation tests. **A**) Epidendrum quitensium, **B**) Sobralia rosea, **C**) Epidendrum anderssonii.

The cryopreservation tests were developed with the seeds of 3 species: *Epidendrum quitensium Rchb.f.*, *Sobralia rosea Poepp.* & *Endl.* and *Epidendrum anderssonii Hágsater* & *Dodson* ( Figure 1). The cryopreservation tests were developed with 3 species:

• 2392 Epidendrum quitensium Rchb.f., (0° 17’52.1"N 78° 22’33.3"W 3200 msnm)
• 2420 Sobralia rosea Poepp.& Endl. (0° 52’11.8"N 78° 26’53.8"W 600 msnm)
• 2706 Epidendrum anderssonii Hágsater&Dodson (0° 50’36.2"N 78° 25’01.5"W 1200 msnm)

The species pertain to three different altitudes and were selected from many sources and have capsules with viable seeds. The seeds collected from the forest were stored in an absorbant paper bag with respective codes for the plant, after they were stored in a Ziplock bag with rice of 12% humidity.

### Freezing speed

Two types of freezing were tested, suggested according to Mroginski *et al* ^7^. The sample units had 0.2 g of seeds stored in cryo tubes (091.11.102, ˙ISOLAB, Wertheim, Germany) of 2 ml. Steps of freezing: freezing was carried out in the following sequence, 0°C for 1 hour by placing the samples in an refrigerator (Electrolux, Stockholm, Sweden), -22°C for 1 hour placing the seeds in a freezer (Selecta Templow, Barcelona, Spain), - 60°C for 1 hour inserting the seeds in an ultra low temperature freezer (New Brunswick Scientific, Edision, NJ, USA), then the seeds were held at 196°C by submerging the samples in liquid nitrogen contained in a thermal container. Finally the samples were placed in racks and stored in a thermal tank (STATEBOURNE biorack 5400, Washington, UK). Rapid freezing: the samples were placed directly in liquid nitrogen at 196°C by immersion using a procedure similar to that used in steps of freezing. In addition, four combinations of cryo preservatives were analyzed: 1- DMSO 1M (Fisher Scientific, Hampton, NH, USA); 2- DMSO 1M (Fisher) – glycerol 1M; 3- DMSO 1M (Fisher) – sucrose 1M; 4-DMSO 1M (Fisher) – glycerol 0.5M – sucrose 0.5M (Fisher) ( Table 1).

**Table 1.** System design and freezing seed symbology used for cryoprotective substances and their concentrations. M: molar.

TYPE OF FREEZING			
GRADUAL (P)		Rapid (F)	
0° ___ -22° ___ -60° ___ -196		-196°	
**CRYOPRESERVANTES**	**SYMBOL**	**CONCENTRATION**	
NONE	N		
DMSO	D	1M	
GLYCEROL	G	1M	0,5M
SUCROSE	S	1M	0,5M
**COMBINATION OF CRYOPRESERVANTES**			
NONE			N
DMSO 1M			D
DMSO 1M	GLYCEROL 1M		DG
DMSO 1M	SUCROSE 1M		DS
DMSO 1M	GLYCEROL 0,5M	SUCROSE 0,5M	DGS

### Seed viability

Seed viability was tested after freezing. Briefly, 5mg of seeds was added to 1.5 ml of 10% sucrose solution and left at 25° C for 24 hours, the seeds were washed with water and 1ml of triphenyl tetrazolium chloride solution (TTC, 1%) (Sigma-Aldrich, St Louis, MI, USA) was added, and then incubated at 40° C for 24 hours. Finally, the seeds were washed with sterilised water and observed under the microscope with a 4x lens (MC100Led, MI-CROS, St. Veit/Glan, Austria). The process for calculating the TTC method was carried out as follows: -Observe the seeds in microscope using lense 4X. -Identify viable seeds and non viable seeds. -Use cross multiplication to determine the average of viability of all seeds.

### Statistical analysis

The experimental design 2x5 with three repetitions was applied to analyse the freezing methods ( Table 2). The results were analyzed by unidirectional ANOVA with 95% confidence. To determine the best treatments the Duncan test was used. This analysis was carried out with RStudio 3.1 (package: Agricolae).

**Table 2.** Experimental design, testing orchid seeds cryopreservation - design 2x5 with three repetitions, Symbols (N: none, D: DMSO, G: glycerol, S: sucrose, P: Freeze steps, R: Rapid).

	STEP (P)			FAST (R)		
	P1	P2	P3	R1	R2	R3
N	PN1	PN2	PN3	RN1	RN2	RN3
D	PD1	PD2	PD3	RD1	RD2	RD3
DG	PDG1	PDG2	PDG3	RDG1	RDG2	RDG3
DS	PDS1	PDS2	PDS3	RDS1	RDS2	RDS3
DGS	PDGS1	PDGS2	PDGS3	RDGS1	RDGS2	RDGS3

## Results

**Figure 2.**
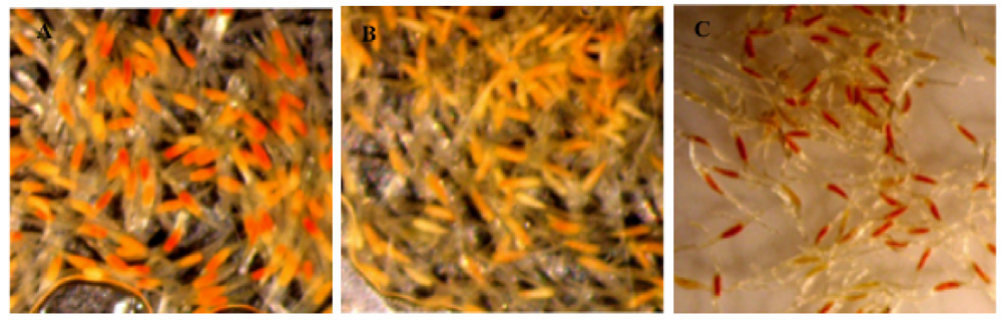
TTC-stained seeds subjected to the "stepped" cryopreservation process without any cryopreservation substances. Viable seeds (dark red embryos) and non-viable (pale embryos). A) Epidendrum quitensium, B) Sobralia rosea, C) Epidendrum anderssonii.

The seeds were considered viable when red coloration of the embryo was observed ^8^ ( Figure 2).

**Figure 3.**
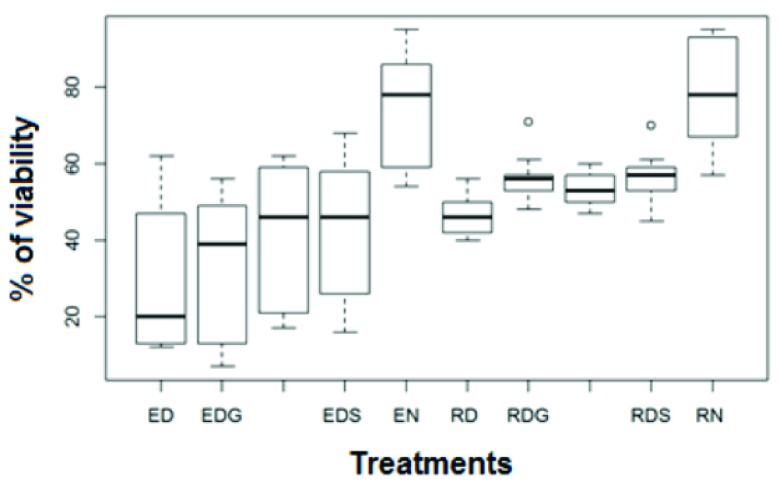
Seed cryopreservation: variability by treatment. Results obtained using the Tukey test.

**Figure 4.**
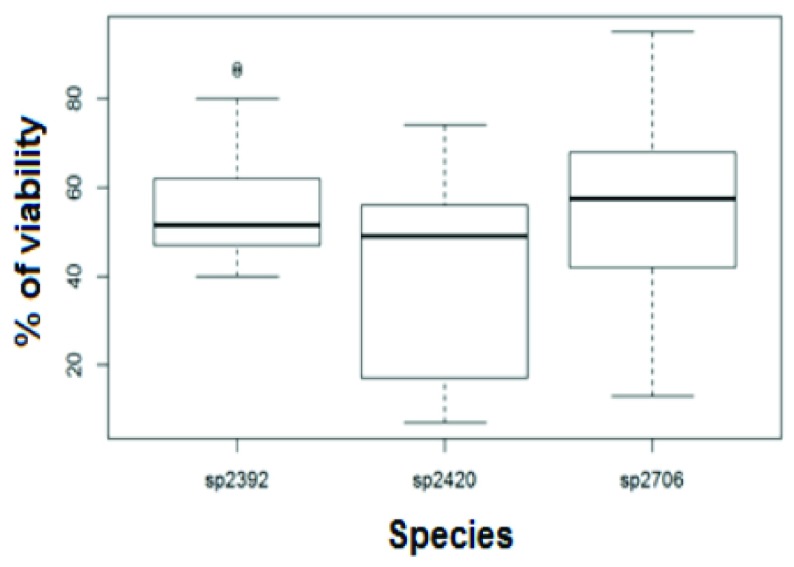
Cryopreservation of seeds: variability by species. Results obtained using the Duncan test, sp2706 (T57.2), sp2392 (T 57.03), sp2420(T39.37).

According to the data obtained ( Table 3, Figure 3), there is a significant difference in the results when comparing the data between the species and between the treatments. According to the Duncan test, the best treatments were rapid freezing and step freezing without the use of cryopreservatives. The least efficient treatment was step freezing with the use of DMSO as a cryopreservant ( Table 4). The species *Epidendrum quitensium* and *Epidendrum anderssonii* showed better results ( Figure 4).

**Table 3.** Cryopreservation of orchid seeds. Values represent percentage of viability assessed by the TTC method, N: cryo preservative; D: DMSO; S: sucrose; G: glycerol.

		Step				
CI	Species	N	D	DG	DS	DGS
2392	*Epidendrum* *quitensium*	83.20	54.87	83.20	46.36	47.01
2420	*Sobralia rosea*	55.93	12.29	10.72	19.34	18.88
2706	*Epidendrum* *anderssonii*	93.50	40.88	51.81	62.84	55.94
		**Rapid**				
2392	*Epidendrum* *quitensium*	74.79	48.29	62.87	52.79	51.73
2420	*Sobralia rosea*	65.60	50.15	55.04	52.72	52.52
2706	*Epidendrum* *anderssonii*	84.49	18.57	41.07	63.08	60.10

**Table 4.** Duncan test groups obtained after cryopreservation test. Results are given as orchid seed viability percentage; a, b, c and d, indicate groups with statistical significance. Classification was made under an alpha of 0.01, and 78 degrees of freedom for error. Symbols (treatment): N: none, D: DMSO, G: glycerol, S: sucrose. Symbols (types of freezing) P: Freeze steps, R: Rapid.

#	Treatment	Mean					
1	RN	78.00	a				
2	PN	74.66	a				
3	RDG	56.66		b			
4	RDS	56.00		b			
5	RDGS	53.33		b	c		
6	RD	46.55		b	c		
7	PDS	43.00			c	d	
8	PDGS	41.88			c	d	
9	PDG	33.33				d	e
10	PD	28.55					e

Dataset 1. TTC-stained seeds subjected to the “Rapid” cryopreservation process: Epidendrum quitensium http://dx.doi.org/10.5256/f1000research.13622.d194233
Copyright: © 2018 Cerna M et al. 2018 Data associated with the article are available under the terms of the Creative Commons Zero "No rights reserved" data waiver (CC0 1.0 Public domain dedication).

Dataset 2. TTC-stained seeds subjected to the “Rapid” cryopreservation process: Sobralia rosea http://dx.doi.org/10.5256/f1000research.13622.d194234
Copyright: © 2018 Cerna M et al. 2018 Data associated with the article are available under the terms of the Creative Commons Zero "No rights reserved" data waiver (CC0 1.0 Public domain dedication).

Dataset 3. TTC-stained seeds subjected to the “Rapid” cryopreservation process: Epidendrum anderssonii http://dx.doi.org/10.5256/f1000research.13622.d194235
Copyright: © 2018 Cerna M et al. 2018 Data associated with the article are available under the terms of the Creative Commons Zero "No rights reserved" data waiver (CC0 1.0 Public domain dedication).

Dataset 4. Percentage for seed viability calculations http://dx.doi.org/10.5256/f1000research.13622.d194236
Copyright: © 2018 Cerna M et al. 2018 Data associated with the article are available under the terms of the Creative Commons Zero "No rights reserved" data waiver (CC0 1.0 Public domain dedication).

## Discussion

Currently, cryopreservation is a safe and cost-effective option for the conservation of endangered species ^9^. In the present investigation, a protocol was developed for cryopreservation of orchid seeds that provides a high percentage of viability, is easy to apply and economical. The seeds of orchids frozen at -196°C can be kept alive with a moisture content of 12% and do not require cryo-protective substances, confirming what is described by Iriondo *et al.* and others ^10, 11^. The use of cryopreservatives is recommended for seeds with a high moisture content, as stated by Reed and others ^12– 14^. Furthermore, Harding ^15^ states that it is necessary to demonstrate the genetic stability of plants regenerated from cryopreserved plant material to approve their release and reintroduction into the environment; but to date, there have been no reports showing changes at the phenotypic, biochemical, chromosomal or molecular levels attributed to storage systems by cryoconservation ^14^. The cryoconservation method that gave the best results was the “Rapid” freezing without the addition of any cryopreservative substance.

## Data availability

The data referenced by this article are under copyright with the following copyright statement: Copyright: © 2018 Cerna M et al.

Data associated with the article are available under the terms of the Creative Commons Zero "No rights reserved" data waiver (CC0 1.0 Public domain dedication). 

Dataset 1: TTC-stained seeds subjected to the “Rapid” cryopreservation process: *Epidendrum quitensium* 10.5256/f1000research.13622.d194233 ^15^

Dataset 2: TTC-stained seeds subjected to the “Rapid” cryopreservation process: *Sobralia rosea* 10.5256/f1000research.13622.d194234 ^16^

Dataset 3: TTC-stained seeds subjected to the “Rapid” cryopreservation process: *Epidendrum anderssonii*. 10.5256/f1000research.13622.d194235 ^17^

Dataset 4: Percentage for seed viability calculations 10.5256/f1000research.13622.d194236 ^18^
