# Supplementary figures and images for: Cryopreservation of orchid seeds through rapid and step freezing methods
Source: F1000Res. 2018 Feb 20;7:209. [Version 1] doi: 10.12688/f1000research.13622.1 (PMC6051192; doi:10.12688/f1000research.13622.1)

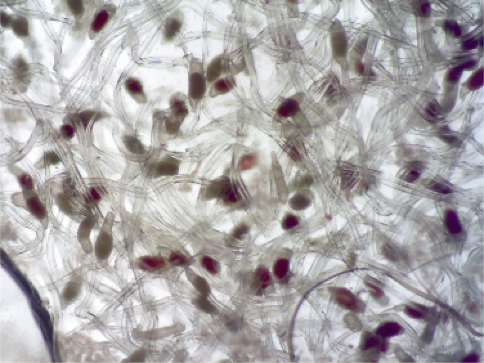

Supplement: Dataset 1. TTC-stained seeds subjected to the “Rapid” cryopreservation process: Epidendrum quitensium — http://dx.doi.org/10.5256/f1000research.13622.d194233 [file f1000research-7-14799-s0000.tgz › 3d5c9e9e-e2ef-4a0c-b5d8-8bb60cfde743_Dataset_1.png]

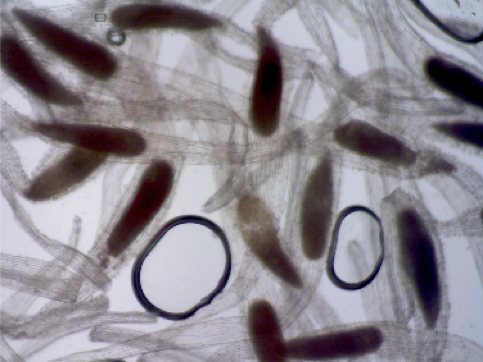

Supplement: Dataset 2. TTC-stained seeds subjected to the “Rapid” cryopreservation process: Sobralia rosea — http://dx.doi.org/10.5256/f1000research.13622.d194234 [file f1000research-7-14799-s0001.tgz › 5c375333-bc08-46b3-bf1c-8fbfe696221c_Dataset_2.png]

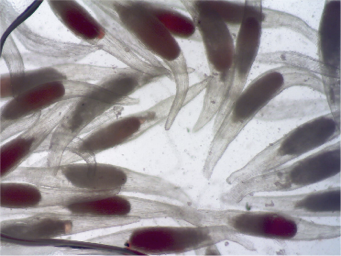

Supplement: Dataset 3. TTC-stained seeds subjected to the “Rapid” cryopreservation process: Epidendrum anderssonii — http://dx.doi.org/10.5256/f1000research.13622.d194235 [file f1000research-7-14799-s0002.tgz › cacadd1b-6a81-4631-8717-a26445c70a09_Dataset_3.png]
