# Supplementary material for: Cryopreservation of orchid seeds through rapid and step freezing methods
Source: F1000Res. 2018 Feb 20;7:209. [Version 1] doi: 10.12688/f1000research.13622.1 (PMC6051192; doi:10.12688/f1000research.13622.1)
Supplement: Peer review history

# Peer review: Referee response for version 1

**DOI:** 10.5256/f1000research.14799.r33998

**Published:** 2018-07-11

**Authors:** Éva Borbélyné Hunyadi

**Affiliations:** 1 Research Institute for Organic Agriculture (ÖMKi), Budapest, Hungary

In the paper entitled "Cryopreservation of orchid seeds through rapid and step freezing methods" written by Marco Cerna *et al*. two types of freezing technique were tested by the seeds of 3 species:

*Epidendrum quitensium Rchb.f., Sobralia rosea Poepp. & Endl. and Epidendrum anderssonii Hágsater & Dodson.*

 The authors present step freezing and rapid freezing methods for cryopreservation purposes. There was a significant difference in the results  between the species and between the treatments. The cryoconservation method that provided the best results was the "Rapid" freezing, without the addition of any substance.

 The introduction is well structured, helping the readers to understand the issue the method tries to solve. Considering that it is a research note the methodology is very detailed.

 Nevertheless, in my opinion it is important to highlight the viability of the developed method on a wider spectrum if possible. In the current form of the Discussion, only general statements can be found regarding the viability of the developed method.

 A more concrete concluding sentence is missing that could answer the following question:

 For what kind of other plants, could this method be useful?

 If the developed method can be applied "only" for orchid seeds, a concluding sentence should be placed in the Discussion, stating the limiting factors (in terms of species) of the method. Although in this study only 3 species were examined, I believe that its important to inform the readers about the authors' recommendation regarding the potential usefulness in other species.

I have read this submission. I believe that I have an appropriate level of expertise to confirm that it is of an acceptable scientific standard.

# Peer review: Referee response for version 1

**DOI:** 10.5256/f1000research.14799.r32906

**Published:** 2018-04-09

**Authors:** Song-Jun Zeng

**Affiliations:** 1 Key Laboratory of South China Agricultural Plant Molecular Analysis and Gene Improvement & Guangdong Provincial Key Laboratory of Applied Botany, South China Botanical Garden, Chinese Academy of Sciences, Guangzhou, China

The authors described a protocol for cryopreservation of seeds of three tropical orchids in liquid nitrogen (LN). They tested rapid and progressive cooling with the controlled temperature at 0°C, -22°C, -60°C and -196°C. They also included the application of three different cryoprotectants such as DMSO, glycerol and sucrose. The viability of tested seeds was checked using tetrazolim test. The protocol might have potential for the cryopreservation of  these orchids. However, the manuscript cannot be accepted to publish at present and need major revision for the following main reasons:.

1. Abstract: Authors could not bring the conclusion ‘Species of the genus *Epidendrum* presented a more efficient response in comparison to *Sobralia*.’, because only one species was be tested in the this manuscript.
2. Introduction: Orchidaceae is a large family with many endangered species and all of them are included in the Convention on International Trade in Endangered Species of Wild Fauna and Flora (CITES) I **and** II.
  And should be revised as ‘or’.
  Current research of orchid Cryopreservation should be introduced in the section.
3. Methods: DPMS/MAE,FloraX the behind of ‘,’ should have a blank.
4. *Epidendrum quitensium **Rchb.f**., Sobralia rosea **Poepp. & Endl**. *and *Epidendrum anderssonii *Hágsater & Dodson
*Rchb.f., Poepp. & Endl.* and *Hágsater & Dodson* should not be italic *.*
5. 2392 **Epidendrum quitensium** Rchb.f., 2420 **Sobralia rosea** Poepp.& Endl., **Epidendrum anderssonii** Hágsater & Dodson
  Epidendrum quitensium, Sobralia rosea, Epidendrum anderssonii should be italic *.*
6. Figure 1: Epidendrum quitensium, Sobralia rosea, Epidendrum anderssonii should be italic *.*
7. Figure 2: A) *Epidendrum quitensium*, B) *Sobralia rosea*, C) *Epidendrum anderssonii*
  A, B, C should be labelled in Photos.
8. Figure 3: What is ED, EDD……what is the meaning of “E”?
9. sp2706 (T57.2), sp2392 (T 57.03), sp2420(T39.37), should be replaced by Latin name of orchids species.
10. Discussion: The discussion should be rewritten. There were few relevant literatures of orchid Cryopreservation were cited in this manuscript. There have lots of related literatures for orchid Cryopreservation at present, which should be discussed and what is the innovation of the manuscript?
11. All the source data should be executed by statistical analysis.

I have read this submission. I believe that I have an appropriate level of expertise to confirm that it is of an acceptable scientific standard, however I have significant reservations, as outlined above.

# Peer review: Referee response for version 1

**DOI:** 10.5256/f1000research.14799.r31828

**Published:** 2018-03-28

**Authors:** Alzbeta Novotna

**Affiliations:** 1 Department of Plant Taxonomy and Nature Conservation, University of Gdańsk, Gdańsk, Poland

The authors describe in the presented manuscript titled “Cryopreservation of orchid seeds through rapid and step freezing methods” a new protocol for cryopreservation of seeds of three tropical orchids in liquid nitrogen (LN). They tested rapid and progressive cooling with the controlled temperature at 0°C, -22°C, -60°C and -196°C. They also included the application of three different cryoprotectants such as DMSO, glycerol and sucrose. The viability of tested seeds was checked using tetrazolim test. Red coloured embryos were considered as the main indicator of the preserved viability of the tested seeds. I suppose that this work brings a novelty to a cryoscience research, especially due to the geographical origin of the studied material. Neotropical Orchidaceae should be prioritized in various research studies for their ecological vulnerability and/or a shortage of knowledge. Especially, the research achievements from such country as Ecuador (the orchid biodiversity hotspot), are important for science. However, numerous modifications and a deep correction of the text should be accomplished.

1. English correction is strongly recommended throughout the whole text. There are many errors in English grammar in the current form of the manuscript, which cause huge difficulties in text reading.
2. Abstract: The authors should be focused on the topic of cryopreservation. The first two sentences in the abstract are out of topic. Moreover, the number of the orchid species (provided number 4032) is not correct. The authors should consider to check the work of D. Neill published in 2015 (¿Cuantas especies nativas de plantas vasculares hay en Ecuador?; Revista Amazónica Ciencia y Tecnología) and Cataloque of the Vascular Plants of Ecuador available on www.tropicos.org, for example.
3. Abstract, sentence “In cryopreservation, temperatures reach below -190°C” should be rephrased because the cryostorage is a long-lasting preserving of studied material at temperature of -80°C, -130°C upto -196°C, which is the temperature of the liquid nitrogen.
4. Abstract, the authors should rephrase the sentence “The present research focuses on……” because of repetition of the verb to focus. Instead of “…for a subsequent generation of a seed bank” to use “for future seed collection” for example.
5. Abstract, “the use of cryo-protective substances”, better to use the term „cryoprotectants“.
6. Introduction; The first half of the abstract is out of the topic of cryopreservation. The authors should be focused on this topic. Moreover, the authors should provide much more citations regarding this topic and discuss shortly the history of cryopreservation of seeds with more interest in orchid seeds. There are many available papers regarding this topic.
7. Methods; The first paragraph should be included in “Acknowledgement” at the end of the paper, not in methods. However, each part of the Methods should be provided with a number, e.g. 1. 1 Collection of the seed material. The sentence “The cryopreservation test were developed with 3 species” is repetition of the first sentence of this paragraph.
8. The authors should use "a.s.l."as the abbreviation for above sea level; the provided abbreviation "msnm" is not understandable. The part of the sentence” The species pertain to three different altitudes….. „ is a repetition of the information written before. Authors write “…..were selected from many sources“, what does it mean? The authors should provide more detailed information about the sources. Then, the part of the sentence …“and have capsules with viable seeds” does not bring the clear information. Were the capsules opened during collection or not? When did the authors collect the seeds (the date)?
9. Methods; Freezing speed. Better to rewrite the title, e.g. Cryopreservation of the seeds
10. This part should be shorten. Besides, the citation of the first sentence Mroginski et al. does not correspond with the number 7. Totally different citation is written under this number. The authors should check the citations of provided literature in the text. Moreover, the authors should provide the information about **duration** of cryostorage. This very important information is missing.
11. In the paragraph “Freezing speed” the authors provide information about placing the tested orchid seeds under the temperature 0°C in a refrigerator. However, this fact is not written in the abstract.
12. The authors should eliminate “…., contained in a thermal container”.
13. The sentence “Finally the samples were places in racks and store in thermal tansk……” should be eliminated, because it is not understandable.
14. Freezing speed; In addition,….should be eliminated because the application of cryoprotectants is one of the main objectives of this research paper. It is not something additional.
15. Why did the authors not test application of sucrose and glycerol individually?
16. The authors should clearly demonstrate, which treatment is considered as a control.
17. Table 1 should be deleted, because all the information given in this table is written in the text.
18. Figure 1; Title should be improved, e.g. “Orchid species used in this study”.
19. The paragraph of “Seed viability” should be eliminated. Instead just one sentence with a citation of the work, where this method was used as first, should be placed at the end of the previous paragraph.
20. Statistical analysis; The authors should explain better of the meaning “The experimental design 2x5”. It is not clear from the written text.
21. Results; The sentence “The seeds were considered viable when red…..” is actually the part of Material and methods.
22. Results; this part should be re-written. The information should be provided more clearly.
23. Table 3; What is CI? Better to use term “Gradual” instead of “step”. The abbreviations of each treatment are already provided in Material and Methods.
24. Figure 3; Authors should provide information about the meaning of “E” (in e.g. ED, EDG) and “R” (in e.g. RD, RDG).
25. Figure 4; Authors should provide information about the meaning of sp2392, sp2420 and sp2706.  It is not clear.
26. Discussion; First, the obtained main result should be provided and discussed with much more available literature. Especially, the authors should compare their findings with achievements in other studies. The provided references are very limited. The works dealing with cryostorage of seeds of other plant families should be included. The given number of cited paper in discussion does not correspond to the number provided in the References.

I have read this submission. I believe that I have an appropriate level of expertise to confirm that it is of an acceptable scientific standard, however I have significant reservations, as outlined above.
